# Disparities in invasive pneumococcal disease, pneumonia, and otitis media among US children by comorbidity profile and insurance status

**DOI:** 10.3389/fpubh.2025.1558157

**Published:** 2025-07-18

**Authors:** Rotem Lapidot, Ahuva Averin, Derek Weycker, Liping Huang, Jeffrey Vietri, Adriano Arguedas, Alejandro Cane, Alexander Lonshteyn, Mark H. Rozenbaum, Stephen I. Pelton

**Affiliations:** ^1^Boston Medical Center, Boston, MA, United States; ^2^Division of Pediatric Infectious Diseases, Rambam Health Care Campus, Haifa, Israel; ^3^Faculty of Medicine, Technion - Israel Institute of Technology, Haifa, Israel; ^4^Boston University Chobanian & Avedisian School of Medicine, Boston, MA, United States; ^5^Avalere Health, Washington, DC, United States; ^6^Pfizer Inc., Collegeville, PA, United States; ^7^Pfizer Inc., Capelle a/d IJssel, Netherlands

**Keywords:** *Streptococcus pneumoniae*, infections, pneumonia, otitis media, child

## Abstract

**Background:**

Near-universal pediatric use of pneumococcal conjugate vaccines in the United States (US) has yielded substantive reductions childhood invasive pneumococcal disease (IPD), pneumonia (PNE), and otitis media (OM), especially among at-risk populations. We evaluated residual disparities in disease burden among US children by comorbidity profile and insurance type (as a proxy for socioeconomic status) during the post-PCV13 era.

**Methods:**

We conducted a retrospective observational cohort study using two US healthcare claims databases: Optum Clinformatics DataMart (commercial) and Merative MarketScan Medicaid Multi-State Database. The two study populations comprised children aged <18 years and were stratified by age and comorbidity profile. Study outcomes included IPD, PNE, OM, and tympanostomy tube (TT) insertion, and were ascertained monthly during the follow-up period. Disease rates were expressed per 100,000 person-years, and age-specific relative rates were calculated by insurance type and comorbidity profile.

**Results:**

Children with comorbidities aged <2 years had the highest rates of IPD and PNE, regardless of insurance status. Rates of IPD and PNE were also higher in children with Medicaid (vs. commercial) insurance; differences generally decreased with increasing age. Differences in incidence of OM and TT insertions between children with (vs. without) comorbidities were absent in the first 2 years of life but became apparent with increasing age.

**Conclusion:**

Children with comorbidities and those with Medicaid insurance have a higher burden of IPD, PNE, and OM. Researchers should assess the impact that preventative strategies have on pediatric populations with the highest rates of disease to identify progress in achieving equity in health.

## Introduction

The incidence of invasive pneumococcal disease (IPD), all-cause pneumonia (PNE), and all-cause otitis media (OM) declined in both fully immunized and under-immunized US children following initiation of universal childhood immunization with 7-valent pneumococcal conjugate vaccine (PCV) in 2000 and 13-valent PCV in 2010 (1–6). IPD rapidly declined from ≥79 cases per 100,000 in children aged <5 years in 1999, to 21 cases per 100,000 by 2009, to 7 cases per 100,000 by 2019 (1). Declines in invasive and non-invasive pneumococcal disease were also reported for high-risk subgroups, including children with specific comorbidities and those from certain socioeconomic backgrounds (3–5).

The burden of invasive and respiratory pneumococcal disease prior to the introduction of PCV was highest in infants, especially preterm and low birth weight infants, and in socioeconomically disadvantaged subgroups (7). While immunization with PCVs has reduced racial and socioeconomic disparities in vaccine-type IPD incidence, differences in disease rates persist presumably due in large part to non-vaccine serotypes (IPD) and/or other pathogens (PNE, OM) (8). A 2014 study of active surveillance data for IPD reported a higher incidence of disease following introduction of PCVs (2001–2009) among Black children versus White children (adjusted rate ratio = 1.6) (9). A 2010 literature review reported socioeconomic status (SES) as the most common identified risk factor for OM and that white children were more likely to undergo tympanostomy tube insertion compared to Black or Hispanic children (10). In the present study, we evaluated whether young children, those with comorbidities, and those who have insurance coverage through Medicaid (as a proxy for low SES) still suffer a disproportionate burden of pneumococcal disease in the late PCV13 era (2015–2019).

## Materials and methods

### Study design and data sources

We conducted a retrospective observational cohort study using data from two healthcare claims databases—the Optum Clinformatics DataMart and the Merative MarketScan Medicaid Multi-State Database. The Clinformatics DataMart captures healthcare claims and enrollment information for >12.5 million plan members annually, including enrollees, their spouses, and their dependents; older adults enrolled in a Medicare Advantage Plan are also included. The MarketScan Medicaid Multi-State Database includes healthcare claims and enrollment information for approximately ten million geographically dispersed enrollees in Medicaid managed care plans or Medicaid fee-for-service plans on an annual basis. For this study, data from each source spanned the period from January 2015 through December 2019. Enrollment in Medicaid was used as a proxy for lower SES status relative to those with commercial insurance since Medicaid eligibility is designated for low-income families and qualified pregnant women and children such as those with disabilities.

Each database comprises medical (i.e., facility and professional service) and outpatient pharmacy claims. Data available from each facility and professional-service claim include dates and places of service, diagnoses, procedures performed/services rendered, and quantity of services (professional-service claims only). Data available for each outpatient pharmacy claim include the drug (class) dispensed, dispensing date, quantity dispensed, and number of days supplied. Selected demographic and eligibility information (including age, sex, geographic region of residence, dates of plan eligibility) is available for all enrollees in the databases. All data can be arrayed to provide a detailed chronology of medical and pharmacy services used by each plan member over time.

### Study population

From each of the two study databases, study populations comprised children who were aged <18 years at any time from January 2015 through December 2019, and were categorized into age-specific subgroups (<1, 1– < 2, 2– < 6, 6– < 12, and 12– < 18 years). For children aged <1 year on their first day of healthcare coverage during this period, the corresponding date was designated the “index date” (Appendix A). For children aged 1–17 years on their first day of healthcare coverage, attention was limited to those with continuous coverage during the subsequent 1-year period (which was employed to ascertain comorbidity profiles); the last date of this period was designated the index date. Because children aged 1– < 2 years were required to have at least 1 year of coverage following their enrollment date, and because this requirement meant that most of these children were aged ≥2 years after their first year of coverage, children in the 1– < 2 years age group at the beginning of follow-up were under-represented. From the Optum Clinformatics DataMart, qualifying children were limited to those with commercial insurance (i.e., those with other/unknown types of insurance were excluded from the commercial subset).

### Study measures

IPD, all-cause PNE, all-cause OM, and TT insertions were ascertained during the follow-up period, which began on the index date and ended on the healthcare coverage end date, age 18 years, or the end of the study period, whichever occurred first. Follow-up was characterized on a monthly basis (i.e., in 30-day intervals), based on healthcare coverage at the beginning of the month.

Study measures were identified using diagnosis, procedure, and drug codes on healthcare claims for inpatient admissions and outpatient encounters (Appendix B,C). All qualifying encounters occurring within 30 days of each other were considered part of the same episode (i.e., qualifying encounters during a single episode may be separated by no more than 30 days). Multiple episodes per patient were thus identified.

### Baseline characteristics

Baseline characteristics included age, sex, and comorbidity profile. Comorbidity profile was defined as “with comorbidities” versus “without comorbidities” based on the presence or absence of conditions that increase the risk of pneumococcal disease, as specified by the US CDC, including asplenia; asthma; cancer; cerebrospinal fluid leak; chronic lung, heart, liver, and kidney disease; diabetes; human immunodeficiency virus; prematurity; sickle cell disease; and use of medications that weaken the immune system (11). In addition, neuromuscular disorders and trisomy 21 were included based on recent publications (12, 13).

Congenital disorders were ascertained using all available healthcare claims data (i.e., before or after index date); chronic medical conditions were ascertained based on healthcare claims information at any time prior to the month of follow-up (i.e., they were defined as time-dependent variables). Operational algorithms (including diagnosis and procedure codes) for identifying comorbidity profiles are available from the authors upon request.

### Statistical analyses

Rates of study measures were reported per 100,000 person-years and were adjusted for differential follow-up using a population-based approach (i.e., total number of episodes divided by total number of person-months of observation, multiplied by 12). Techniques of nonparametric bootstrapping (with replacement) were used to characterize 95% confidence intervals (CI) for rates and corresponding relative rates. Analyses were conducted separately for commercial-insured children and Medicaid-insured children (i.e., study populations were not combined into a single analytic file); for each study population, analyses were conducted by age, and within each age-specific subgroup, by comorbidity profile.

### Data availability

Study data sources are proprietary, provided by a third-party vendor, and the authors do not have permission to disseminate the data without vendor approval.

## Results

### Baseline characteristics

From the Optum Clinformatics DataMart, a total of 4.9 million children aged <18 years qualified for inclusion in the “Commercial” study population (Supplementary Table 1). The percentage of children by age group was: <1 year, 21%; 1– < 2 years, <1% (low % due to study design; see Study Population); 2– < 6 years, 17%; 6– < 12 years, 30%; and 12– < 18 years, 31%. The prevalence of ≥1 comorbidity across age groups ranged from 2.8% (age <1 year) to 4.5% (age 6- < 12 years) (Table 1; Supplementary Table 2).

**Table 1:** Baseline characteristics of children aged <18 years in commercial and Medicaid study populations.

	Commercial study population, by age (years)					Medicaid study population, by age (years)				
	<1	≥1 – < 2	≥2 – < 6	≥6 – < 12	≥12 – < 18	<1	≥1 – < 2	≥2 – < 6	≥6 – < 12	≥12 – < 18
	(*N* = 1,047,246)	(*N* = 18,785)	(*N* = 843,663)	(*N* = 1,444,563)	(*N* = 1,519,915)	(*N* = 2,249,637)	(*N* = 275,024)	(*N* = 2,145,266)	(*N* = 2,913,590)	(*N* = 2,478,062)
Demographic profile										
Age (years), mean (SD)	0.0 (0.0)	1.0 (0.0)	3.6 (1.1)	8.5 (1.7)	14.5 (1.7)	0.0 (0.0)	1.0 (0.0)	3.5 (1.1)	8.4 (1.7)	14.4 (1.7)
Sex										
Male	51.4	50.5	51.3	51.1	51.0	51.2	50.7	51.4	51.5	51.2
Female	48.6	49.5	48.7	48.9	49.0	48.8	49.3	48.6	48.5	48.8
Comorbidity profile										
Without comorbidities	97.2	96.4	95.6	95.5	95.8	93.9	92.7	93.4	93.1	93.9
With comorbidities	2.8	3.6	4.4	4.5	4.2	6.1	7.3	6.6	6.9	6.1
Year of follow-up start, %										
2015	27.1	100.0	39.4	45.6	46.3	33.9	100.0	57.1	60.2	56.4
2016	19.1	0.0	20.9	19.2	19.0	17.2	0.0	15.0	15.1	18.1
2017	18.8	0.0	10.7	9.4	9.2	17.5	0.0	10.6	9.7	10.3
2018	18.1	0.0	14.4	12.9	12.7	14.6	0.0	8.5	7.4	7.3
2019	17.0	0.0	14.6	12.9	12.9	16.8	0.0	8.8	7.7	7.9

SD: standard deviation.

From the MarketScan Medicaid Multi-State Database, a total of 10.1 million children aged <18 years qualified for inclusion in the “Medicaid” study population. The percentage of children by age group was: <1 year, 22%; 1– < 2 years, 3%; 2– < 6 years, 21%; 6– < 12 years, 29%; and 12– < 18 years, 25%. The prevalence of ≥1 comorbidity across age groups ranged from 6.1% (age <1 year) to 7.3% (age 1– < 2 years). Asthma, prematurity, and cardiac disease were the most prevalent comorbidities in both commercial-insured and Medicaid-insured children; the prevalence of comorbidities was generally higher among children with Medicaid insurance across age groups.

### Rates of pneumococcal disease

**Figure 1:**
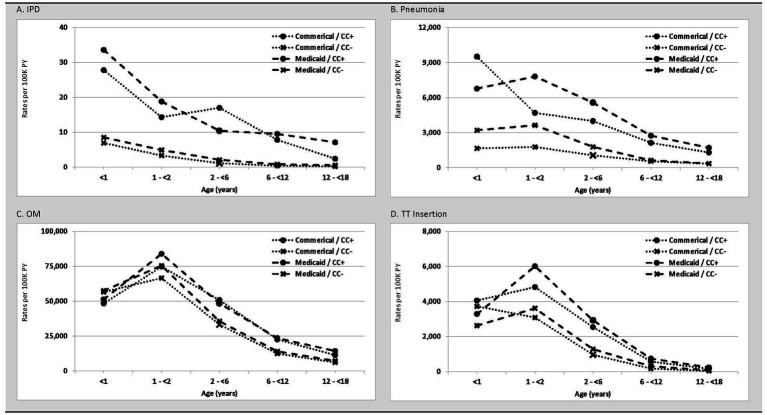
Rates (per 100 K PY) of **(A)** IPD, **(B)** Pneumonia, **(C)** Otitis Media, and **(D)** Tympanostomy Tube Insertion in commercial and Medicaid study populations, by age and comorbidity profile. CC+, children with comorbid conditions; CC-, children without comorbid conditions; PY, person-years.

IPD rates were highest in the first year of life and declined with increasing age regardless of insurance coverage and comorbidity profile (Figure 1; Table 2). The greatest burden of IPD, PNE, and OM were found in children with comorbidities. Relative rates of IPD for Medicaid-insured versus commercial-insured children ranged from 1.3 (95% CI: 0.9–1.9; infants <1 year) to 3.4 (95% CI: 1.5–7.1; children 12– < 18 years). Relative rates of IPD were ~4-fold higher among infants aged <2 years with comorbidities versus those without comorbidities among both commercial- and Medicaid-insured populations. Relative rates (i.e., with vs. without comorbidities) increased to >10-fold higher in children aged >6 years. Although disease incidence declined rapidly with increasing age in both insured groups and among children with and without comorbid conditions, the excess burden of IPD in children with comorbid conditions was present throughout childhood.

**Table 2:** Rates of invasive pneumococcal disease and pneumonia in commercial and Medicaid study populations, by age and comorbidity profile.

Age/insurance type	No. of child-years	Invasive pneumococcal disease			Pneumonia		
		Rates (95% CI) per 100 K PY	Rel. Rate (95% CI)		Rates (95% CI) per 100 K PY	Rel. Rate (95% CI)	
			CC + vs. CC-	MDCD vs. Comm*		CC + vs. CC-	MDCD vs. Comm*
<1 Year							
Commercial	630,761	7.5 (5.6–9.9)			1,881 (1,847–1,915)		
CC+	17,980	27.8 (11.6–66.8)	4.1 (1.3–10.2)		9,510 (9,070–9,972)	5.7 (5.5–6.0)	
CC-	612,781	6.9 (5.1–9.3)			1,657 (1,625–1,689)		
Medicaid	1,652,964	10.0 (8.6–11.6)		1.3 (0.9–1.9)	3,411 (3,383–3,440)		1.8 (1.8–1.9)
CC+	95,301	33.6 (23.7–47.5)	3.9 (2.6–5.8)	1.2 (0.3–4.0)	6,768 (6,605–6,935)	2.1 (2.1–2.2)	0.7 (0.7–0.8)
CC-	1,557,664	8.5 (7.2–10.1)		1.2 (0.8–1.8)	3,206 (3,178–3,234)		1.9 (1.9–2.0)
1– < 2 Years							
Commercial	522,177	3.6 (2.3–5.7)			1,846 (1,810–1,884)		
CC+	14,001	14.3 (3.6–57.1)	4.3 (0.5–18.0)		4,693 (4,347–5,065)	2.7 (2.4–2.9)	
CC-	508,177	3.3 (2.1–5.4)			1,768 (1,732–1,805)		
Medicaid	1,317,478	5.8 (4.6–7.2)		1.6 (0.9–2.8)	3,879 (3,846–3,913)		2.1 (2.1–2.1)
CC+	79,832	18.8 (11.3–31.2)	3.8 (2.0–6.8)	1.3 (0.1–11.9)	7,805 (7,614–8,001)	2.2 (2.1–2.2)	1.7 (1.5–1.8)
CC-	1,237,646	4.9 (3.8–6.3)		1.5 (0.8–2.7)	3,626 (3,593–3,660)		2.1 (2.0–2.1)
2– < 6 Years							
Commercial	1,727,581	1.7 (1.2–2.4)			1,166 (1,150–1,183)		
CC+	64,568	17.0 (9.4–30.8)	15.7 (6.7–35.2)		3,990 (3,838–4,147)	3.8 (3.6–3.9)	
CC-	1,663,013	1.1 (0.7–1.7)			1,057 (1,041–1,073)		
Medicaid	4,810,524	2.7 (2.3–3.3)		1.6 (1.0–2.5)	2,045 (2,032–2,058)		1.8 (1.7–1.8)
CC+	337,916	10.4 (7.4–14.4)	4.8 (3.1–7.1)	0.6 (0.3–1.3)	5,568 (5,489–5,648)	3.1 (3.1–3.2)	1.4 (1.3–1.5)
CC-	4,472,608	2.2 (1.8–2.6)		2.0 (1.1–3.5)	1,779 (1,767–1,791)		1.7 (1.7–1.7)
6– < 12 Years							
Commercial	3,326,947	0.8 (0.5–1.1)			616 (608–625)		
CC+	153,214	7.8 (4.4–13.8)	17.8 (7.5–41.4)		2,130 (2,059–2,205)	3.9 (3.8–4.1)	
CC-	3,173,733	0.4 (0.3–0.7)			543 (535–551)		
Medicaid	7,389,426	1.5 (1.2–1.8)		1.9 (1.2–3.1)	794 (788–801)		1.3 (1.3–1.3)
CC+	592,519	9.5 (7.3–12.3)	11.7 (7.9–17.3)	1.2 (0.6–2.5)	2,741 (2,699–2,783)	4.4 (4.3–4.5)	1.3 (1.2–1.3)
CC-	6,796,908	0.8 (0.6–1.1)		1.8 (0.9–3.6)	624 (619–630)		1.2 (1.1–1.2)
12– < 18 Years							
Commercial	3,783,914	0.3 (0.2–0.5)			381 (375–388)		
CC+	164,676	2.4 (0.9–6.5)	12.6 (2.7–49.4)		1,297 (1,243–1,353)	3.8 (3.6–4.0)	
CC-	3,619,238	0.2 (0.1–0.4)			340 (334–346)		
Medicaid	6,430,442	1.0 (0.8–1.3)		3.4 (1.5–7.1)	451 (446–456)		1.2 (1.2–1.2)
CC+	464,070	7.1 (5.1–10.0)	14.1 (8.3–24.1)	2.9 (0.6–11.4)	1,701 (1,664–1,739)	4.8 (4.7–4.9)	1.3 (1.2–1.4)
CC-	5,966,372	0.5 (0.4–0.7)		2.6 (0.9–7.0)	354 (349–359)		1.0 (1.0–1.1)

CC-: children without a comorbid condition; CC+: children with ≥1 comorbid condition; Comm: commercial; MDCD: Medicaid; PY: person-years.

*Relative rates for Medicaid vs. commercial, overall and within subgroups defined on comorbidity profile.

PNE incidence was highest during the first 2 years of life and declined monotonically thereafter among all subgroups defined on insurance type and comorbidity profile. The incidence of PNE ranged from 1,657 (95% CI: 1,625–1,689; commercial-insured without comorbidity) and 9,510 (95% CI: 9,070–9,972; commercial-insured with comorbidity) per 100,000 child-years in year 1 of life, and from 1,768 (95% CI: 1,732–1,805; commercial-insured without comorbidity) and 7,805 (95% CI: 7,614–8,001; Medicaid-insured with comorbidity) in the second year of life. Relative rates of PNE among commercial-insured infants aged <1 year and those aged 1– < 2 years with comorbidities versus those without comorbidity were 5.7 [95% CI: 5.5–6.0] and 2.7 [95% CI: 2.4–2.9], respectively, and those insured by Medicaid (vs. commercial-insured) had higher relative rates of PNE (1.8 [95% CI: 1.8–1.9] and 2.1 [95% CI: 2.1–2.1]), respectively. Relative rates for those with comorbidities were more modest compared with IPD.

Peak rates of OM occurred in year 2 of life and decreased progressively thereafter (Table 3). Approximately 50% of infants had an OM episode in year 1 of life, and nearly 75% of children between 1 and 2 years of age had an episode. Unlike IPD and PNE, differences in OM rates between children with comorbidities versus without comorbidities were minor in the first 2 years of life and progressively increased thereafter to nearly 2-fold higher by age 12– < 18 years. Minor differences in rates were also observed between commercial-insured and Medicaid-insured children within age cohorts.

**Table 3:** Rates of otitis media and tympanostomy tube insertion in commercial and Medicaid study populations, by age and comorbidity profile.

Age/insurance type	No. of child-years	Otitis media			Tympanostomy tube insertion		
		Rates (95% CI) per 100 K PY	Rel. Rate (95% CI)		Rates (95% CI) per 100 K PY	Rel. Rate (95% CI)	
			CC + vs. CC-	MDCD vs. Comm*		CC + vs. CC-	MDCD vs. Comm*
<1 Year							
Commercial	630,761	56,311 (56,126–56,496)			3,731 (3,683–3,779)		
CC+	17,980	48,308 (47,303–49,335)	0.9 (0.8–0.9)		4,049 (3,765–4,354)	1.1 (1.0–1.2)	
CC-	612,781	56,545 (56,357–56,734)			3,721 (3,673–3,770)		
Medicaid	1,652,964	57,115 (57,000–57,230)		1.0 (1.0–1.0)	2,657 (2,632–2,682)		0.7 (0.7–0.7)
CC+	95,301	51,328 (50,875–51,785)	0.9 (0.9–0.9)	1.1 (1.0–1.1)	3,282 (3,169–3,399)	1.3 (1.2–1.3)	0.8 (0.7–0.9)
CC-	1,557,664	57,469 (57,350–57,588)		1.0 (1.0–1.0)	2,618 (2,593–2,644)		0.7 (0.7–0.7)
1– < 2 Years							
Commercial	522,177	66,807 (66,586–67,029)			3,129 (3,081–3,177)		
CC+	14,001	74,738 (73,320–76,184)	1.1 (1.1–1.1)		4,821 (4,471–5,199)	1.6 (1.4–1.7)	
CC-	508,177	66,588 (66,364–66,813)			3,082 (3,034–3,131)		
Medicaid	1,317,478	75,916 (75,768–76,065)		1.1 (1.1–1.1)	3,751 (3,718–3,784)		1.2 (1.2–1.2)
CC+	79,832	83,920 (83,287–84,558)	1.1 (1.1–1.1)	1.1 (1.1–1.1)	6,011 (5,844–6,184)	1.7 (1.6–1.7)	1.2 (1.1–1.4)
CC-	1,237,646	75,400 (75,247–75,553)		1.1 (1.1–1.1)	3,605 (3,572–3,639)		1.2 (1.1–1.2)
2– < 6 Years							
Commercial	1,727,581	33,885 (33,799–33,972)			1,018 (1,003–1,033)		
CC+	64,568	50,784 (50,237–51,337)	1.5 (1.5–1.5)		2,535 (2,415–2,661)	2.6 (2.5–2.8)	
CC-	1,663,013	33,229 (33,142–33,317)			959 (945–974)		
Medicaid	4,810,524	36,475 (36,421–36,529)		1.1 (1.1–1.1)	1,393 (1,383–1,404)		1.4 (1.3–1.4)
CC+	337,916	48,453 (48,219–48,689)	1.4 (1.4–1.4)	1.0 (0.9–1.0)	2,928 (2,871–2,986)	2.3 (2.2–2.3)	1.2 (1.1–1.2)
CC-	4,472,608	35,570 (35,515–35,626)		1.1 (1.1–1.1)	1,277 (1,267–1,288)		1.3 (1.3–1.4)
6– < 12 Years							
Commercial	3,326,947	12,939 (12,900–12,977)			185 (181–190)		
CC+	153,214	22,587 (22,351–22,827)	1.8 (1.8–1.8)		565 (529–604)	3.4 (3.2–3.6)	
CC-	3,173,733	12,473 (12,434–12,512)			167 (162–171)		
Medicaid	7,389,426	14,810 (14,782–14,838)		1.1 (1.1–1.1)	333 (329–337)		1.8 (1.7–1.9)
CC+	592,519	23,505 (23,382–23,629)	1.7 (1.7–1.7)	1.0 (1.0–1.1)	735 (713–757)	2.5 (2.4–2.5)	1.3 (1.2–1.4)
CC-	6,796,908	14,052 (14,024–14,080)		1.1 (1.1–1.1)	298 (294–302)		1.8 (1.7–1.8)
12– < 18 Years							
Commercial	3,783,914	6,305 (6,280–6,330)			43 (41–45)		
CC+	164,676	11,331 (11,170–11,495)	1.9 (1.8–1.9)		156 (138–176)	4.1 (3.6–4.7)	
CC-	3,619,238	6,076 (6,051–6,102)			38 (36–40)		
Medicaid	6,430,442	7,713 (7,692–7,735)		1.2 (1.2–1.2)	81 (78–83)		1.9 (1.8–2.0)
CC+	464,070	14,269 (14,160–14,378)	2.0 (2.0–2.0)	1.3 (1.2–1.3)	234 (221–248)	3.4 (3.2–3.7)	1.5 (1.3–1.7)
CC-	5,966,372	7,203 (7,182–7,225)		1.2 (1.2–1.2)	69 (66–71)		1.8 (1.7–1.9)

CC-: children without a comorbid condition; CC+: children with ≥1 comorbid condition; Comm: commercial; MDCD: Medicaid; PY: person-years.

*Relative rates for Medicaid vs. commercial, overall and within subgroups defined on comorbidity profile.

TT insertions were most common in the first two years of life with rapid decline after age 6 years. Except for the first year of life, rates of TT insertion were modestly higher among children with Medicaid (vs. commercial) insurance. Infants and children with (vs. without) comorbidities had higher rates of TT insertion, with relative differences between these cohorts increasing with age.

## Discussion

This study evaluated the incidence of IPD, PNE, and OM among children aged <18 years between 2015 and 2019, a time period following a 90% decline in childhood IPD and a shift from disease primarily due to PCV13 serotypes to non-PCV13 serotypes (8, 14, 15). We selected this time period, 2015–2019, as the impact of PCV13 on IPD appeared to stabilize based on data from the Active Bacterial Core surveillance system (1). We evaluated disease burden by age, comorbidity status and insurance status as a proxy for SES.

We found the greatest incidence of IPD in infants aged <1 year, regardless of insurance status, with progressive decline with increasing age. Using Medicaid insurance as a proxy for SES, IPD incidence was higher in Medicaid-insured children among cohorts with and without comorbidities, indicating that disparities based on SES persist in the PCV13 era. The relative rate of IPD for Medicaid-insured children increased with increasing age, reflecting a more rapid decline of IPD with age in children with commercial insurance. The observation that the burden of IPD is greater among persons living in higher-poverty census tracts is well described (16). The persistence of a greater IPD burden among children insured by Medicaid most likely reflects that although the disparity in incidence of vaccine serotype disease has narrowed, differences persist for non-vaccine serotype disease. This observation is consistent with Raman’s report that non-vaccine serotype IPD remained highest in neighborhoods that are most deprived (17, 18). However, disparities in immunization rates among children insured by Medicaid may also contribute to the observed increased disease burden (19–21).

Children and adults with asthma, chronic heart disease, or chronic lung disease are recognized to be at greater risk for pneumococcal disease (22, 23). Although these comorbidities are not linked to specific immune deficits, it has been hypothesized that chronic inflammation may compromise host defenses such as the respiratory barrier increasing susceptibility to pneumococcal disease (24). Our analyses also found that higher rates of IPD among infants and children with comorbidities have persisted despite high rates of completion of the primary PCV13 regimen (25).

Our analysis reveals that the burden of PNE and OM remains high, especially in children less than 2 years of age, and is not equally shared by all children. Children with, compared to without, comorbid conditions have a greater incidence of disease throughout childhood. We also found that IPD and PNE were more common among children with Medicaid insurance, while the difference in the incidence of OM was minimal between children with different insurance coverage.

For PNE, we observed that peak incidence occurred during the first two years of life, and that incidence was higher in children with (vs. without) comorbidities and those with Medicaid (vs. commercial) insurance. Differences in PNE rates by comorbidity profile persisted across age groups. The incidence of PNE in children insured by Medicaid was also greater for all age groups; however, unlike IPD, the difference diminished with increasing age.

For OM, incidence rates peaked in the second year of life. Unlike PNE and IPD, only minor differences were observed in the incidence of OM and TT insertion between children grouped by insurance or comorbidity status early in life. These findings vary from those of a 2010 literature review which reported SES as a common risk factor for OM (10). We hypothesize that this finding reflects Medicaid expansion among lower SES children (26), which likely improved access to care during our study period (i.e., compared with previously published studies) and could have changed healthcare seeking behavior among lower-income parents. Beginning after age 2 years, children with comorbidities had higher rates of OM and a progressive increase in relative risk. In children without comorbidities, OM rates declined rapidly after age 2 years; in children with comorbidities, the decrease was modest until age 6 years and thereafter the decay was slower than in healthy children. We demonstrate that the burden of OM remains high, specifically in the first two years of life, in both children with and without comorbidity. The data also identify older children with comorbidity as an at-risk group for OM.

The incidence of TT insertions reflected the burden of OM and the priority for restoration of hearing in children with comorbidities consistent with clinical practice guidelines (27). Both episodes of OM and TT insertion decline after 6 years of age (28, 29). The two broadest indications for TT insertion are recurrent acute OM (RAOM) and OM with persistent effusion (OME) (30). Published studies vary regarding the impact of race and socioeconomic status on RAOM and OME. A recent publication reports that social deprivation, characterized by the social deprivation index, was associated with lower likelihood of treatment for recurrent and suppurative OM and lower odds of TT placement (31). Our study did not find differential rates of TT insertion based on insurance status, which although not identical to social deprivation index, also reflects socioeconomic disadvantage. The aforementioned study covered 2003 to 2021 compared with our data set, which reflects 2015–2019. It is possible that the more recent data reflect progress in reducing disparity in access to TT insertion between children with divergent insurance status; however, our data does not permit assessment of change over an extended time period.

We note several limitations to the research described herein. Rates of PNE among children with asthma (included in the cohort with comorbidity) could be upwardly biased as atelectasis or clinical symptoms assigned could be misclassified as PNE in such children. However, IPD is known to be more frequent in children with asthma supporting the likelihood that pneumococcal pneumonia is also more likely in children with asthma, consistent with our findings for children with comorbid conditions (22). The same is true for children with prematurity; IPD is documented to have higher incidence, and other investigators have also reported increased rates of PNE (32). We are unaware if potential bias (e.g., over-diagnosis of atelectasis as PNE in children with asthma) would affect otitis media as well; asthma has been reported as risk factor for OM by other investigators (33, 34).

Algorithms for identifying study measures have not been formally evaluated against a “gold standard” and thus their accuracy is unknown. Similarly, use of operational algorithms to characterize comorbidity profiles undoubtedly resulted in misclassification of some children. Because children aged 1–17 years were required to have at least 1 year of continuous healthcare coverage for purposes of characterizing baseline comorbidity profiles, rates of study outcomes during this period were not considered. For this same reason (i.e., requiring 1 year of coverage before tracking outcomes), children aged 1– < 2 years at the beginning of their follow-up were under-represented in the study population.

In addition, because date of birth is not available in the study data source (except for the subset of children for whom their birth hospitalization is captured in the data source), age was determined using year of birth (and assuming a birth date of July 1) or the healthcare coverage effective date (i.e., if coverage effective date is during birth year). Accordingly, for some children, age may be mis-specified. While comparisons of study measures between commercial-insured and Medicaid-insured children were largely limited to subgroups defined on age and comorbidity profile, to the extent insurance-specific subgroups differ based on other risk factors, comparisons may be confounded. Because SES information is not available in the study data sources, type of insurance coverage (i.e., commercial vs. Medicaid) was used as a proxy. Immunization history could not be accurately ascertained as we suspect that many children received vaccines through the Vaccines for Children program, which is not reflected in the study databases. We note, however, that recent US CDC data (2019–2020 births) show that uptake of all recommended vaccines among children aged <24 months was 69.1% and uptake of PCVs (≥4 doses) was 82.7%, though there were statistically significant differences in vaccine uptake for commercial- and Medicaid-insured infants (19). Finally, while the study databases include information on many patients across demographic profiles and geographic regions, caution should be used in generalizing study results to other populations and settings.

In summary, despite declines in IPD, PNE, OM and TT insertion, the burden of these clinical syndromes remains high and not equally shared by all children. Two to 4 % of children are diagnosed with PNE by age 2 years. More than 50% are diagnosed with AOM, and 3–6% have TT insertion in each of the first two years of life. For IPD and PNE, children with comorbidities and those insured by Medicaid suffer a disproportional burden of disease. For OM and TT insertions, our analysis reveals that the usual pattern of declining incidence after age 2 years differs among children with comorbidities, in whom incidence decreases more slowly, and a disproportionate burden of disease persists. Researchers should assess the impact that preventative strategies have on pediatric populations with the highest rates of disease to identify progress in achieving equity in health.

## Data Availability

The data analyzed in this study is subject to the following licenses/restrictions: study data sources are proprietary, provided by a third-party vendor, and the authors do not have permission to disseminate the data without vendor approval. Requests to access these datasets should be directed to the data vendors Optum and Merative.
